# Nanobodies as Versatile Tool for Multiscale Imaging Modalities

**DOI:** 10.3390/biom10121695

**Published:** 2020-12-18

**Authors:** Marco Erreni, Tilo Schorn, Francesca D’Autilia, Andrea Doni

**Affiliations:** Unit of Advanced Optical Microscopy (HAOUM), Humanitas Clinical and Research Center—IRCCS, 20089 Rozzano (MI), Italy; tilo.schorn@humanitasresearch.it (T.S.); francesca.dautilia@humanitasresearch.it (F.D.)

**Keywords:** nanobody, imaging, microscopy

## Abstract

Molecular imaging is constantly growing in different areas of preclinical biomedical research. Several imaging methods have been developed and are continuously updated for both in vivo and in vitro applications, in order to increase the information about the structure, localization and function of molecules involved in physiology and disease. Along with these progresses, there is a continuous need for improving labeling strategies. In the last decades, the single domain antigen-binding fragments nanobodies (Nbs) emerged as important molecular imaging probes. Indeed, their small size (~15 kDa), high stability, affinity and modularity represent desirable features for imaging applications, providing higher tissue penetration, rapid targeting, increased spatial resolution and fast clearance. Accordingly, several Nb-based probes have been generated and applied to a variety of imaging modalities, ranging from in vivo and in vitro preclinical imaging to super-resolution microscopy. In this review, we will provide an overview of the state-of-the-art regarding the use of Nbs in several imaging modalities, underlining their extreme versatility and their enormous potential in targeting molecules and cells of interest in both preclinical and clinical studies.

## 1. Introduction

Molecular imaging has emerged as a key tool for the visualization of biological processes associated to a variety of diseases. In the last decades, remarkable progresses have been done in the field of molecular imaging, with an increasing number of new technologies aimed at characterizing, with increased accuracy and sensitivity, biological processes in vitro and in vivo [1,2,3,4,5]. Several imaging modalities, having different features and peculiarities, have been developed, in order to provide structural and functional information related to the progression of different pathologies. However, no single imaging modality has the ability to provide details about all the aspects of a specific biological event. Non-invasive in vivo imaging technologies, such as positron emission tomography (PET), single-photon emission tomography (SPECT), optical imaging (OI), magnetic resonance imaging (MRI) and ultrasound provide spatial and temporal information about the anatomical localization and metabolic status of biological processes in living animals: however, they lack the possibility to analyze events at cellular or sub-cellular resolution, a limitation that can be solved through the use of intravital or ex vivo microscopy technologies. For this reason, the use of multiple imaging modalities has become preferred [6,7,8].

In the last decades, a new class of heavy chain-only antibodies derived from Camelidae, referred to as nanobodies (Nbs), has gained a growing interest in the field of imaging, given their peculiar features and high versatility [9,10,11,12].

In this review, we will provide a general overview of Nb characteristics and their in vivo application for the molecular imaging investigation of biological processes involved in pathologies. Subsequently, we will discuss their recent application in in vitro sub-cellular microscopy and super-resolution techniques.

## 2. Nanobodies: Characteristic and Structure

Non-invasive in vivo imaging techniques, including PET, SPECT, OI and ultrasound, require the accumulation of specific probes targeting the tissue of interest, in order to achieve a high contrast signal compared to surrounding healthy tissue [13]. In addition, imaging tracers have to be biocompatible, stable and non-toxic. In order to increase the specific accumulation to the targeted tissue, imaging probes are generally couple to molecules like antibodies, small ligands or peptides [14,15,16,17,18,19,20]. In this context, specificity of monoclonal antibodies (mAbs) is considered an important feature and, indeed, they represent a widely used tool in several molecular imaging protocols. However, their high immunogenicity due to a species-specific response, heterotetrameric composition and large size (150 kDa) result in longer blood circulation time and reduced tissue penetration, thus limiting their effectiveness as imaging probes [21]. To overcome this issue, several antibody fragments and variants have been generated, maintaining the antibody typical features, but having a significantly lower molecular weight. Several molecules, including Fabs, single chain Fv (sc-Fv) and minibodies, have been developed: although these antibody fragments are smaller than conventional antibodies (molecular weight ranging from 25–100 kDa), thus showing higher tissue penetration, they are prone to denaturation, spontaneous dimerization and the formation of immunogenic aggregates [22,23,24,25].

The discovery of particular homodimeric heavy chain-only antibodies (HCAbs) in camelids leads to the development of a third generation of antigen binding fragments, referred to as nanobodies (Nbs) [10]. Nbs represent the variable domain of the HCAbs heavy chain (VHH) and are defined as the smallest natural antigen binding fragment derived from the camelid heavy chain-only antibodies [26]. Compared to whole antibody, as well as their derived fragments, Nbs are much smaller, with an approximate molecular weight of 12–15 kDa, therefore suggesting decreased problems related to steric hindrance in molecular recognition. From the structural point of view, Nbs are similar to human variable heavy chain (VH), comprising 2 β-sheets, with 4 and 5 β-strands, having 3 different binding sites. Moreover, differently from human VH, Nbs are extremely soluble and resistant to aggregation [27,28]. In addition, their reduced size provides several advantages in terms of biological activity: (I) Nbs are weakly immunogenic in humans, as a consequence of the absence of the most immunogenic portion of Fc and their high similarity with the human type 3 VH domain [29,30]; (II) Nbs show an excellent tissue penetration capability and a rapid blood clearance, mostly by renal elimination: while conventional monoclonal Abs show a residence time in blood ranging from few days to weeks in humans, Nbs are generally cleared within few hours, resulting in a significantly increased signal to noise ratio [31,32,33,34,35,36]; (III) Nbs are demonstrated to have efficient folding/refolding capacity and high stability, even under unfavorable conditions of pH, pressure, temperature and protease exposure [37,38,39]; (IV) Nbs are easy to clone and produce, since they can be synthesized by microbial system and do not require post-translational modifications [11]; (V) although Nbs have only three complementary-determining regions (CDR), against the 6 of conventional antibodies, they show a similar antigen affinity. CDRs play a crucial role in Nb stability and binding affinity, as their peculiar conformation increases the variety of recognized epitopes. In addition, Nb CDR3 is longer than human VHs, allowing the possibility to bind antigens located deeper in the target cavities and slits, including ion channels, G protein-coupled receptors and immune synapses [33,37,40,41,42,43,44,45].

Another key feature of Nbs is their high modularity, allowing the possibility to be engineered to generate bivalent Nbs (2 Nbs connected by a peptide linker, to increase their avidity), biparatopic Nbs (2 connected Nbs recognizing 2 different epitopes of the same antigen) and bispecific Nbs (connecting 2 Nbs recognizing different antigens). In addition, as well as for whole antibodies, Nbs can be linked to a broad range of molecules, including nanoparticles, viruses, drugs and imaging probes [46,47,48].

## 3. Generation of Nanobody Libraries

A variety of methods are currently available for the production of Nbs, generally involving the generation of a Nb library and the following selection of the best candidates [49,50].

Briefly, three types of Nb banks can be used to obtain antigen-specific Nbs, namely immune, naïve and synthetic library. The vast majority of Nbs described to date are obtained by selections of phage libraries displaying the variable domains of HCAbs derived from immunized camels. In the immune library, healthy Bactrian camel, dromedary, llama or alpaca are immunized with the target antigen. Over 2 months, animals are injected multiple times: in addition, since animals are outbred and they generate single immune responses, usually more than one animal is immunized [41]. After immunization, B cells are isolated from blood, mRNA is extracted and then converted in cDNA, which is finally used to amplify the VHH region. Subsequently, amplicons are cloned in phagemid vectors, the library is phage-displayed and subjected to consecutive rounds of biopanning on recombinant proteins or cells. Positive clones are then inserted in appropriate expression vectors for the production of Nbs in microbial hosts, such as *E. coli, S. cerevisae* or *P. pastoris* [51,52,53]. Nbs produced by bacteria are then extracted by osmotic shock, sonication or freeze-thaw cycles [54]. In addition, yeast or mammalian cells are able to secrete Nbs in the supernatant, ensuring the correct formation of possibly required post-translational modifications [55].

Anyway, in some cases, the generation of an immune library is difficult to achieve. Some molecules, including DNA or RNA, are not immunogenic or at least fail to induce an HCAb-mediated immune response, while other antigens might be too toxic or pathogenic. In these cases, the generation of naïve or synthetic Nb library might be considered [56,57,58]. Naïve library is generated starting from a larger amount of blood collected from healthy, non-immunized animals. High-affinity binders are then selected by biopanning procedure to identify best candidates. Although several reports demonstrated the possibility to generate high affinity Nbs from naïve library, the large amount of blood (>10 L) necessary to obtain 10^10^ VHH clone might represent a limitation [31,59,60]. Alternatively, the generation of synthetic libraries may be convenient [50]. To this aim, a stable Nb scaffold is selected, mainly based on biochemical properties, including stability, high-expression level and solubility. Subsequently, the codons of amino acids located in the antigen-binding loops are randomized and amplified by polymerase chain reaction (PCR). The PCR products are then inserted in an appropriate vector and used to transform *E. coli,* to finally generate the library [61].

Although different approaches have been used to generate antigen-specific Nb libraries, immune libraries have a much higher high-affinity Nb title, compared to naïve and synthetic libraries, making easier to obtain functional Nbs [62]. In addition, the main advantage of library display technology is the ability to direct the selection process towards specific properties, such as the resistance to protease [63] or the ability to migrate through the blood brain barrier [31].

In addition to immunizing a camel, several transgenic mice, able to produce HCAbs, have been developed and successfully used to generate Nbs, especially in cases where the antigen was difficult to obtain [64,65]. These transgenic mice contain a hybrid llama-human antibody locus, generated by recombining two variable llama regions and the human D, J, Cμ and /or Cγ constant regions.

## 4. Nanobodies as Imaging Tools

Nbs have emerged as important tools for both in vitro and in vivo imaging modalities. Due to their small size (around 15 kDa) and the absence of Fc fragment, Nbs are rapidly eliminated from the circulation and penetrate more easily into target tissue, resulting in a significantly reduced background and increased signal-to-noise ratio [13,66].

Several imaging techniques have been successfully optimized for Nb applications. Due to their sensitivity, quantitative output and clinical relevance, the majority of in vivo molecular imaging studies have been performed using PET and SPECT, labeling Nbs with positron-emitting nuclides (^18^F, ^68^Ga or ^89^Zr) and γ-emitting nuclide (^99m^Tc), respectively [67,68]. In ultrasound imaging, Nbs have been tagged to contrast agents, such as microbubbles and nanobubbles and mainly used for the visualization of the systemic vasculature [69]. For OI, Nbs are tagged with fluorescent dyes and detected by non-invasive in vivo imaging modality, intravital microscopy and ex vivo flow-cytometry [35,54]. In addition, optical imaging is used to visualize surface lesions during endoscopic examinations or in the setting of fluorescence-guided surgery [68]. Moreover, due to their capacity to fold in reducing environment, Nbs are suited for the expression in the cytosol of mammalian cells, allowing their application in tracking intracellular proteins by conventional and super-resolution microscopy [70,71].

**Figure 1:**
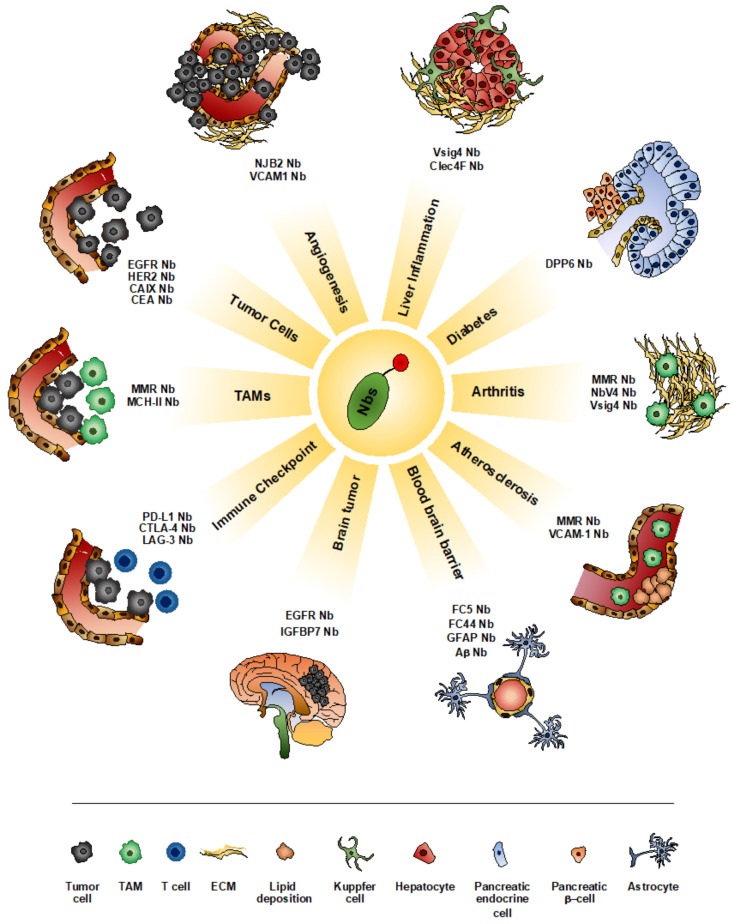
Nanobodies (Nbs) as imaging tool for the visualization of different pathological conditions. Nbs have been successfully used for the visualization of a variety of pathologies. Different aspects of neoplastic progression have been evaluated using Nbs targeting tumor cells (EGFR-Nb, HER2-Nb, CAIX-Nb and CEA-Nb), tumor angiogenesis (NJB2-Nb and VCAM-1-Nb), TAM infiltration (MMR-Nb and MHC-II-Nb) or components of immune checkpoints (PD-L1-Nb, CTLA-4-Nb and LAG-3-Nb). Similarly, several inflammatory diseases have been successfully monitored using Nbs, including liver inflammation (Vsig-Nb and ClecF4-Nb), diabetes (DPP6-Nb), rheumatoid arthritis (MMR-Nb, NbV4-Nb and Vsig-Nb) and atherosclerosis (MMR-Nb and VCAM-1-Nb). Moreover, Nbs have been applied for the visualization of brain tumors (EGFR-Nb and IGFBP7-Nb). In addition, due to their small dimension, Nbs more easily cross the blood brain barrier and accumulate into the brain parenchyma (FC5-Nb, FC44-Nb, GFAP-Nb and Aβ-Nb).

Due to their intriguing features, Nbs have been proposed as a potential molecular imaging agent for the visualization of different pathological conditions, including inflammation, atherosclerosis, central nervous system (CNS) diseases and tumors [47,72,73,74] (Figure 1).

### 4.1. Imaging Tumor

**Figure 2:**
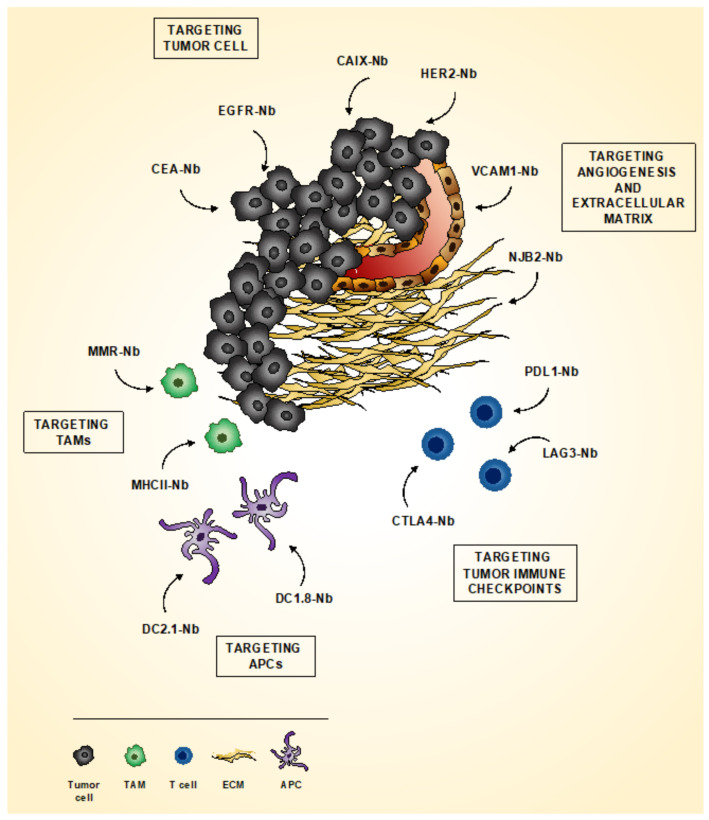
Imaging tumor with Nbs. In the last decades, several Nbs have been generated to detect different components and cell populations of the tumor microenvironment in vivo. Nbs directed against HER2, CAIX, EGFR and CEA have been widely used, alone or in combination to visualize neoplastic cells. Tumor angiogenesis and extracellular matrix (ECM) composition have been investigated using anti-VCAM-1 and anti-NJB2 Nbs. Moreover, pro-tumorigenic tumor-associated macrophages (TAMs) have been identified by anti-MMR Nbs, while TAMs associated to anti-tumor response have been detected by anti-MHC-II Nbs. Other Nb-based probes have also been designed to target antigen presenting cells (APCs). Finally, several Nbs have been developed for immune-checkpoint inhibitor, including CTLA-4 and PD-L1. More recently, anti-LAG3 Nbs have been generated to detect tumor-infiltrating lymphocytes.

Neoplastic tissue represents a complex environment, where cancer cells, surrounding stroma and infiltrating-immune cells cooperate, to establish tumor-promoting conditions. As a consequence, it is becoming increasingly important not only to detect the presence of specific tumor antigens but also to visualize microenvironmental factors relevant for cancer progression, in order to provide an efficient diagnosis and to plan and monitor the most effective treatments (Figure 2).

#### 4.1.1. Imaging Tumor Cells

Nbs are widely used for the direct detection of cancer cells (Figure 1 and Figure 2). Currently, the most advanced Nb probe target is represented by the human epidermal growth factor receptor type 2 (HER2), a member of HER-kinase family, over-expressed by a variety of tumor types, including breast, ovarian, prostate and colorectal cancer [75]. In 2016, a phase I clinical trial demonstrated that a ^68^Ga-HER2 Nbs 2Rs15d was able to detect primary tumor and distant metastases in breast cancer patients already 60 min post-injection, without any adverse effects [76,77]. These results led to a phase II clinical trial, aimed at the identification of brain metastases (NCT03924466). Accordingly, it has been recently demonstrated that anti-HER2 Nbs 2Rs15d successfully detect HER2-positive brain lesion in an orthotopic preclinical model [78]. In addition, a combination of anti-carbonic anhydrase IX (CAIX) and anti-HER2 Nbs has been used for the optical molecular imaging visualization of an orthotopic mouse model of breast cancer, mimicking in situ ductal carcinoma. The anti-CAIX/anti-HER2 Nb synergy significantly increase tumor-to-background ratio, also enabling the detection of small metastasis in the lung [79].

Other members of the HER-kinase family, such as HER1, HER3 and HER4, have been demonstrated to be successfully targeted by Nbs in different tumor models [80]. In particular, HER-1 (called epidermal growth factor receptor, EGFR) targeting Nbs were radiolabeled with ^99m^Tc, showing a rapid clearance and allowing a marked discrimination between EGFR over-expressing and EFGR low-expressing tumors. Similarly, anti-EGFR Nb ^99m^Tc-7C12 was able to monitor the response to tyrosine kinase inhibitor Erlotinib in an A431-tumor xenograft mouse model, using pinhole SPECT/micro-CT. Of note, a reduction in the renal retention and thus potential nephrotoxicity, of ^99m^Tc-7C12 was observed in mice treated with lysine and/or gelofusine, a known inhibitor of megalin, a multiligand binding receptor involved in the tubular reabsorption and retention of Nbs in the kidney [81,82]. Anti-EGFR Nbs 7D12 have also been used, conjugated with the infra-red fluorophore IRDye800CW, for the optical molecular imaging visualization of human tumor xenograft mouse model, showing a rapid and homogeneous distribution throughout the tumor [83,84].

Carcinoembryonic antigen (CEA) is a glycosylated macromolecule, highly expressed in many tumor types, such as lung, breast and colorectal cancer. In the last decades, CEA has been widely considered as an interesting target for cancer diagnosis and therapy [85]. Anti-CEA Nbs, radiolabeled with ^99m^Tc, has been successfully used for the visualization of a mouse model of CEA-positive human colon adenocarcinoma. A major limitation for this radiotracer is represented by the high liver and kidney uptake, which significantly limits the detection of neoplastic lesions close or inside these organs [13]. More recently, IRDye 800CW-conjugated anti-CEA Nbs have been used for tumor visualization in an orthotopic mouse model of pancreatic cancer: anti-CEA Nbs rapidly accumulated in neoplastic lesions, allowing discrimination between high- and low-CEA expressing tumors [86].

This evidence therefore suggests the use of Nbs to visualize different molecules, directly expressed by cancer cells, involved in tumor proliferation and growth.

#### 4.1.2. Imaging Tumor Microenvironment

The tumor microenvironment has a crucial role in cancer development [87]. Extracellular matrix (ECM) components and different cell types, including fibroblasts, endothelial cells (ECs) and immune cells, play a multifaceted role during neoplastic progression, influencing tumor growth and resistance to therapy [88].

The ECM forms the major component of the tumor microenvironment, providing bio-mechanical support, regulating angiogenesis and controlling cancer cell survival and invasion [89]. Recently, a series of Nb libraries against ECM proteins have been developed [90]. In particular, an ECM-specific Nb, NJB2, has been demonstrated to specifically recognize the alternatively spliced EIIIB (EDB) domain of the glycoprotein fibronectin, a major component of tumor ECM and neovasculature [91]: administration of NJB2 results in the detection of both primary tumors and metastatic lesions by immunofluorescence and non-invasive immuno-PET/CT in multiple models of breast cancer and melanoma.

Besides the ECM, the visualization of newly forming blood vessels has been proposed as an interesting tool for the evaluation of anti-angiogenic therapies [92]. Imaging tumor angiogenesis has been performed targeting vasculature cell adhesion molecule-1 (VCAM-1), a protein involved in the adhesion of lymphocytes, monocytes, eosinophils and basophils to the vascular endothelium [93]. Anti-VCAM-1 Nbs have been used to tag microbubbles and applied for the visualization of tumor vasculature by ultrasound imaging: contrast-enhanced ultrasound imaging showed an increased echo signal intensity in cancer tissue already 10 min after VCAM-1 Nb-coated microbubbles compared to control [69].

Inflammatory cells and mediators are key components of the cancer microenvironment [94]. Tumor-associated macrophages (TAMs) represent the most abundant immune infiltrating cell population in tumor tissues, contributing to neoplastic progression by promoting genetic instability, supporting metastatic processes and taming adaptive immunity [95]. Tumor promoting TAMs highly express macrophage mannose receptor (MMR), a transmembrane glycoprotein involved in their scavenging and phagocytic activity [96]. ^99m^Tc-conjugated anti-MMR Nbs have been developed and used for the visualization of mouse model of mammary adenocarcinoma and Lewis lung carcinoma by SPECT/CT [97]: anti-MMR Nbs selectively identified MMR^+^ TAMs infiltrating hypoxic regions, with a high tumor-to-background ratio. Of note, ^99m^Tc-labeled anti-MMR Nbs showed a marked accumulation in the liver and spleen of tumor-bearing mice, which has been minimized by the co-injection of a molar excess of unlabeled bivalent anti-MMR Nbs, without altering tumor uptake [97]. Similarly, anti-MMR Nbs have been labeled with ^18^F and used for the detection of MMR^+^ TAMs by PET in a mouse model of Lewis lung carcinoma, showing a clear tumor accumulation and a reduced liver and kidney uptake [98]. More recently, [^68^Ga]Ga-NOTA-anti-MMR Nbs have been generated, tested in preclinical tumor mouse models and finally proposed for application in a phase I clinical trial [99].

Opposite to MMR, the expression of major histocompatibility complex class II (MHC-II) on macrophages is associated to anti-tumor response and antigen presentation to CD4^+^ T cells: Nbs targeting MHC-II were labeled with ^18^F and successfully applied for the detection of mouse model of pancreatic cancer by immuno-PET/CT, showing a good tumor-to-background ratio [100]. Other Nb-based probes have been designed to target antigen presenting cells (APCs): SPECT imaging of ^99m^Tc-labeled DC2.1 and DC1.8 Nbs revealed a specific recognition of the biodistribution of myeloid cells and immature dendritic cells (DCs), respectively, suggesting their potential application for the targeting of tumor-associated DCs [101].

Altogether, these results indicate the possibility to use Nbs to effectively investigate several components of the tumor microenvironment.

#### 4.1.3. Imaging Tumor Immune Checkpoint

Cancer progression is often associated with immune suppression, as cancer cells are able to activate different immune checkpoint pathways to suppress anti-tumor responses [102]. Since immune checkpoint inhibitors (ICIs) have been shown to be promising tools in immuno-oncology, several Nbs have been developed for the ICI visualization. Monoclonal antibody-based immunotherapies antagonizing the interaction between programmed death-ligand 1 (PD-L1) and its receptor (PD-1) have revolutionized anti-cancer therapy [103,104]. Accordingly, anti-PD-L1, ^99m^Tc-labeled, Nbs have been generated and successfully used to visualize PD-L1 expression in syngeneic tumors in mice by SPECT/CT [105]. In addition, Nbs targeting human PD-L1 have been developed for both SPECT and PET applications and enrolled in a phase I clinical trial in non-small cell lung cancer patients [106,107]. More recently, anti-PD-L1 Nbs have been conjugated with ^68^Ga and proposed as promising PET imaging agent for the clinical assessment of PD-L1 expression in human patients [108].

Cytotoxic T-Lymphocyte-associated protein 4 (CTLA-4) is another well characterized receptor protein, acting as immune checkpoint, which has been widely investigated for immunotherapy. Anti-CTLA-4 Nbs have been generated and used for the visualization of CTLA-4 in a melanoma mouse model [109].

Of note, the first anti-LAG3 (Lymphocyte-Activation gene 3) Nb has been recently developed [110]. LAG-3 is known to downregulate T-cell response and its expression has been often observed on tumor-infiltrating lymphocytes, correlating with shorter disease-free survival in patients treated with anti-PD-1 therapy [111]. ^99m^Tc-labeled anti-LAG3 Nbs were visualized by SPECT/CT and showed specific uptake in immune peripheral organs as well as in tumors [110].

These data indicate the possibility to use Nbs to analyze the contribution of several immune-checkpoint inhibitors in tumor progression.

#### 4.1.4. Theranostic Approach Using Nbs

Most of the Nbs described for their application in tumor imaging have been also investigated for their potential use as theranostic agents, thus combining therapeutic and diagnostic strategy in the same agent. Nbs targeting cancer specific antigen have been effectively used for both imaging and therapeutic purpose: anti-CD20 Nbs was radiolabeled with ^68^Ga and ^177^Lu and used, respectively, for tumor detection, by PET imaging, and targeted therapy in a mouse model of non-Hodgkin lymphoma [112]. Similarly, Nbs binding the monoclonal-protein (M-protein), expressed by myeloma cells, have been generated and labeled with ^99^Tc and ^177^Lu for the SPECT imaging and therapeutic application, respectively, in a multiple myeloma mouse model [113]. More recently, it has been demonstrated that ^131^I-conjugated, anti-HER2 Nbs can be used at low doses, for SPECT imaging, and at higher doses, for targeted radionuclide therapy, to both detect and treat HER2^+^ tumor mouse models [114].

Other Nb-theranostic approaches combine optical imaging and photodynamic therapy: fluorescence-labeled Nbs can be used to identify neoplastic lesion in a setting of fluorescence-guided surgery and, subsequently, residual cells can be eliminated by light activation of Nb-photosensitizer-conjugates [115].

Moreover, Nbs targeting ICIs, including CTLA-4 and PD-L1, have been used to visualize, by PET or SPECT imaging, the activation of immune-suppressive pathway and then administered, alone or in combination with inflammatory molecules, to restore an effective anti-tumor immune response [116,117,118,119].

All these data suggest the possibility to use Nbs as anti-tumor theranostic agents, taking advantage of their peculiar properties.

### 4.2. Imaging Inflammatory Diseases

The high affinity, specificity, solubility and stability, together with small size, increased tissue penetration and rapid blood clearance make Nbs particularly performant tracers for molecular imaging of inflammation (Figure 1 and Table 1) [72].

Atherosclerosis is defined as a progressive narrowing of arteries, as a result of fat deposits and continuous inflammation on their inner lining. Due to the limited dimensions of the atherosclerotic lesions and the proximity to the unbound blood circulating imaging tracers, it is quite challenging to obtain an optimal signal-to-background ratio, to correctly visualize signals associated with vascular plaques. Taking advantage of their small size and rapid blood clearance, Nbs have been largely investigated as potential imaging agent for the non- or minimally-invasive detection of atherosclerotic lesions [72]. Hypercholesterolemia results in an increased expression of VCAM-1 by endothelial cells during atheroma formation [120]: in line with this observation, Nbs targeting VCAM-1 have been used for the in vivo detection of atherosclerotic lesions. In hypercholesterolemic ApoE-deficient mice, ^99m^Tc-labeled anti-VCAM-1 Nb accumulation within the atherosclerotic lesions has been imaged by SPECT [121]. Similarly, Nbs targeting VCAM-1 were conjugated to microbubbles and used for the contrast-enhanced ultrasound molecular imaging of aortic lesions in a mouse model of atherosclerosis [122]. Interestingly, the same approach has been used ex vivo on human endarterectomy specimens, with anti-VCAM-1 Nbs showing increased attachment, compared to control. This observation paves the way for the clinical translation of Nb-based contrast-enhanced ultrasound molecular imaging of early events in atherosclerosis development [122]. More recently, anti-VCAM-1 Nbs were functionalized with the restrained complex agent (RESCA) chelator, conjugated with ^18^F-AIF and used for the visualization of atherosclerotic plaques in ApoE-deficient mice by PET/CT [123]. Of note, using a high-performance preclinical PET imager, it has been possible to detect atherosclerotic lesions with high sensitivity and a sub-millimeter spatial resolution [123].

The formation of atherosclerotic plaques is characterized by the accumulation of macrophages: in particular, MMR^+^ macrophages infiltration has been correlated with neovascularization and intraplaque hemorrhage [124]. In line with this observation, ^99m^TC-anti-MMR Nbs were injected in ApoE-deficient mouse model of atherosclerosis: of note, no significant correlation was found between plaque burden and Nb uptake, probably due to the diffuse distribution of MMR-positive cells in myocardial tissue and along healthy vessels [125]. Interestingly, in the same mouse model, a significant ^68^Ga-NOTA-anti-MMR Nb accumulation was observed, by PET/CT imaging, in the aortic tissue of *ApoE^-/-^* mice, compared to controls [126]. This discrepancy could be explained by the shorter half-life of ^68^Ga compared to ^99m^Tc, which results in a faster blood clearance and better detection of anti-MMR Nbs, together with the higher sensitivity and spatial resolution of PET, compared to SPECT.

Further Nb-based tracers are currently under investigation for their use in other inflammatory diseases. In rheumatoid arthritis (RA), a chronic inflammatory disease of the joints associated by abundant in situ recruitment of immune cells [127], macrophage infiltration was assessed by SPECT/microCT imaging, evaluating the accumulation of ^99m^Tc-labelled anti-MMR Nbs in a mouse model of collagen-induced arthritis (CIA) [128]. Similarly, ^99m^Tc-NbV4m119 Nbs were used to visualize the up-regulation of complement receptor of the Ig superfamily (CRIg), expressed by synovial macrophages, by SPECT/CT, in a mouse model of CIA [129]. Of note, accumulation of ^99m^Tc-NbV4m119 Nbs in the knee of RA mice could be detected before the macroscopic manifestation of the symptoms related to the disease [130]. In addition, in a transient model of sterile inflammatory arthritis, ^99m^Tc-NbV4m119 Nbs allowed the visualization of the onset, progression and resolution of arthritis symptoms [130]. RA synovial macrophages also expressed the type I transmembrane protein V-set and Ig domain-containing 4 (Vsig4): near-infrared fluorescence (NIRF) imaging of Cy7-labeled anti-Vsig4 Nbs showed the accumulation of Vsig4^+^ macrophages in the joint of CIA mice. Moreover, detected signal correlated with the severity of the disease [131].

In addition, it has been demonstrated that anti-Vsig4 Nbs selectively targets Kupffer cells (KCs), liver resident macrophages involved in the controlling of tissue homeostasis and whose presence can vary in different pathological condition. In a in vivo model of concanavalin (ConA)-induced acute hepatitis, a down-regulation of SPECT signal of ^99m^TC-labeled anti-Vsig4 Nbs reflected a reduction in the number of KCs, indicating that Nbs targeting Vsig4 can be used as molecular marker to monitor KC dynamic modulation during hepatic inflammation [132]. In addition to Vsig4, Nbs targeting C-type lectin domain family 4 member F (Clec4F) have been used to monitor KC dynamic during liver inflammation by SPECT: ^99m^TC-labeled anti-Clec4F Nb accumulation is reduced in a ConA-induced acute hepatitis model. On the contrary, induction of steatohepatitis resulted in a higher signal of both ^99m^TC-labeled anti-Vsig4 and ^99m^TC-labeled anti-Clec4F Nbs, corresponding to an increased density of KCs in inflamed liver tissues [133].

Recently, dipeptidyl-Peptidase 6 (DPP6) was identified as a target to evaluate the endocrine cell mass in vivo, for the visualization of the progressive β-cell loss in diabetes or after islet transplantation [134]. Accordingly, a radiolabeled Nb-based tracer targeting DPP6 has been generated and used for the in vivo SPECT/CT imaging of insulin-producing human EndoC- βH1 cells, transplanted in immune-deficient mice [134]. More recently, the same Nb was used to detect different amount of human pancreatic islets following implantation in immune-deficient mice, by both SPECT and PET imaging [135], evidence which emphasize the importance of using Nbs in the assessment of inflammation in different contexts (Table 1).

**Table 1:** Overview of Nb-based tracers for molecular imaging of inflammatory diseases.

Nanobody	Inflammatory Disease Model	Imaging Modality	Reference
Anti-VCAM-1	Atherosclerosis	SPECT, PET/CT, Ultrasound	[121,122,123]
Anti-MMR	Atherosclerosis	PET/CT, SPECT	[125,126]
Anti-MMR	Rheumatoid Arthritis	SPECT/CT	[128]
Anti-CRIg	Rheumatoid Arthritis, Sterile Inflammatory Arthritis	SPECT/CT	[129,130]
Anti-Vsig4	Rheumatoid Arthritis	NIRF-optical imaging	[131]
Anti-Vsig4	Acute Hepatitis, Steatohepatitis	SPECT	[132,133]
Anti-Clec4F	Acute Hepatitis, Steatohepatitis	SPECT	[132,133]
Anti-DPP6	Diabetes	SPECT/CT, PET	[134,135]

### 4.3. Imaging Pathologies of the Central Nervous System (CNS) 

The detection of intracerebral target by molecular imaging probes has always been challenging, mainly due to the presence of the blood-brain barrier (BBB), which acts as a gatekeeper to maintain brain homeostasis [136]. The most investigated process for the delivery of molecules into the brain is via receptor-mediated transcytosis (RMT), a natural system used to shuttle macromolecules, such as vitamins, proteins and nutrients, between blood circulation and brain [137]. In the RMT process, the ligand binds to its receptor, expressed on the luminal surface of endothelial cells (ECs), triggering the internalization of the receptor-ligand complex. Then, the internalized material fuses with abluminal surface of ECs and the ligand is finally released into the brain parenchyma [137].

Two Nbs, FC5 and FC44, have been isolated for their ability to cross the BBB in RMT-mediated process [31]. In particular, FC5 Nbs have been used to coat doxorubicin-loaded, lipid-based nanoparticles, expressing a near-infrared dye on their surface and visualized by in vivo optical imaging. After i.v. injection in mice, FC5-coated nanoparticles delivered into the brain parenchyma were higher compared to the non-targeted control [138].

In addition to RMT, transmigration of macromolecules across the BBB can occur via a non-specific, charge-based, endocytic process upon interaction with the membrane of ECs [139]. Accordingly, it has been demonstrated that some Nbs, with a basic isoelectric point, were able to cross the BBB in vivo after systemic injection, without the need of any additional procedure: specifically, basic Nbs targeting human glial fibrillary acidic protein (GFAP), upon intracarotid and intravenous injection, were able to transmigrate across the BBB, diffuse into the brain parenchyma and bind to GFAP expressing astrocytes [140].

Amyloid beta peptide (Aβ) accumulation in the brain represents an early event in the pathogenesis of Alzheimer’s disease, a form of dementia associated with the formation of plaques and tangles in the brain [141]. Recently, Nbs targeting Aβ (anti-Aβ Nb) and neurofibrillary tangles (anti-tau) have been designed and used for the detection of amyloid plaques in a mouse model of Alzheimer’s disease. 2-photon intravital microscopy showed that, after intravenous administration, anti-Aβ and anti-tau Nbs were able to penetrate the BBB and recognize their target [142]. Of note, in addition to the basic isoelectric point, the smaller dimension of Nbs seems to facilitate their penetration through the BBB, as dimer or EGFP-linked Nbs showed a decreased transmigration [140]. More recently, ^99m^Tc-labeled Nbs targeting amyloidogenic peptides were used to visualize amyloidogenic gelsolin deposition by SPECT/CT, in a mouse model of gelsolin amyloidosis [143].

In the context of brain tumor, such as glioblastomas, the analysis of tumor angiogenesis is a crucial parameter to assess the severity of the disease and, hence, relevant in the choice of the appropriate therapeutic interventions [144]. EGFR and its mutated variant EGFRvIII are over-expressed in 50% of glioblastoma multiforme: engineered versions of anti-EGFR and anti-EGFRvIII Nbs, modified to increase their valency and circulation half-life, have been successfully applied for the in vivo optical imaging of brain tumor in an orthotopic mouse model [145]. Several other tumor vascular targets, such as vascular endothelial growth factor receptor 2 (VEGFR-2), αvβ3 and αvβ5 integrins and insulin-like growth factor binding protein 7 (IGFBP7), have been used to visualize tumor angiogenesis by PET, optical imaging and MRI [74,146,147,148]. In preclinical mouse model of glioblastoma, fluorescent-labeled Nbs targeting IGFBP7 have been applied to visualize tumor vasculature by non-invasive optical imaging [148]. In addition, anti-IGFBP7 Nbs were used to functionalize both Gadolinium (Gd)-coated lipid nanoparticles and superparamagnetic iron oxide nanoparticles for the MRI imaging of tumor angiogenesis [149,150]: of note, bio-conjugation of those nanoparticles with Cy5.5 dye allowed the development of a bimodal optical-MRI in vivo imaging contrast agent.

Altogether, these data clearly suggest the use of Nbs for imaging CNS diseases, being able to highlight tumor angiogenesis and to effectively cross the BBB.

## 5. Nanobodies in Fluorescence-Guided Surgery

Real-time intraoperative guidance is an essential tool to support surgeons during the resection of neoplastic tissues. Recently, fluorescence-guided surgery (FGS) has begun to be considered an innovative approach for surgical navigation [115,151]. In 2011, the first FGS application has been performed in patients with ovarian cancer, consisting in the administration of fluorescein isothiocyanate (FITC)-conjugated folic acid during cyto-reductive surgery [152]. However, due to tissue autofluorescence occurring at visible wavelengths, associated with reduced light penetration during illumination and fluorescence scattering, surgical settings resulted to be sub-optimal. Indeed, for in vivo imaging application, the choice of fluorophores emitting in the near-infrared range of wavelength (between 680 nm to 900 nm) is mandatory, as reduced scattering, limited tissue absorption and less autofluorescence intrinsically improve light tissue penetration, spatial resolution and signal-to-background ratio [153]. In addition, the proper selection of the dye is crucial, since it has been demonstrated that fluorophore conjugation significantly impact on the pharmacokinetic of the tracer [154]. Intraoperative surgery guidance in solid tumors remains a medical need and more recent methodologies based on parallel detection of different fluorescence parameters (use of specific fluorescent probes and intrinsic lifetimes) have been proposed to in vivo visualize tumor margins and factors typically associated to regions of potential invasiveness (increase of anaerobic glycolysis and acidic pH) [155].

Several Nbs have been recently evaluated for their application as imaging tracers in FGS [68]. Due to their small dimensions, the impact of dye conjugation on Nb pharmacokinetic is significant [35]: it has been demonstrated that IRDye800-CW labeling results in an atypical Nb biodistribution, with higher background signal and liver accumulation [156]. Similarly, IRDy680RD or AF680 labeling shows lower background but conjugated-Nbs are partially secreted via the hepatobiliary route [157]. In addition, the chemistry of conjugation seems to be determining too, influencing tumor targeting, uptake specificity and clearance [156]. Finally, multiple labeling of Nbs is not advisable, as the close proximity of the dyes, due to the small dimension of the Nbs, could result in the quenching of the fluorescent signal.

In the context of FGS, several Nbs have been developed. IRDye800CW-labeled anti-EGFR Nbs were used to delineate orthotopic oral squamous cell carcinoma and cervical lymph node metastasis: 24 h after Nb administration, neoplastic lesions were clearly identified and resected [158]. Previously, we have reported the use of Nbs targeting HER-2 and CAIX for the visualization of mouse model of breast cancer: similarly, fluorescent-conjugated anti-HER2 and anti-CAIX Nbs were applied in an experimental setup mimicking intra-operative settings [159,160]. In particular, combination of anti-HER2 and anti-CAIX Nbs resulted in an increased tumor contrast, enabling the identification of small metastasis in the lung [79]. In addition, in a transplanted model of ovarian cancer, IRDye800CW-anti-HER-2 Nbs were used for the identification and surgical removal of tumor nodule within the abdomen with a submillimeter resolution [161]. More recently, anti-CEA Nbs, conjugated with the near-infrared dye IRDye800CW, were successfully used for FGS in an orthotopic transplanted model of pancreatic cancer [86]: fluorescent anti-CEA Nbs rapidly and precisely accumulated in tumor tissue, showing a labeling kinetic that was similar to the one of non-specific tracers, such as indocyanine green. More interesting, the Nb fluorescent signal has been detected using 2 clinical imaging devices already approved by the Food and Drug Administration, thus paving the way for the clinical translation of Nb-based FGS in human surgery.

## 6. Nanobodies in Microscopy

**Figure 3:**
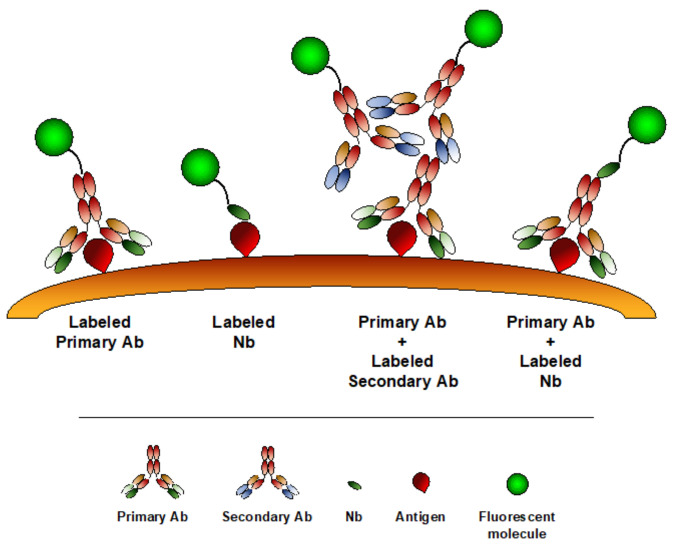
Nbs minimize the “linkage error.” Due to their smaller dimension compared to conventional Abs, the use of Nbs significantly reduces the distance between the antigen and the fluorophore, thus minimizing the so-called “linkage error.”.

Optical microscopy represents a widely used imaging modality to analyze processes at cellular and subcellular levels, preserving the sample physiological conditions. In the last years, enormous progresses have been made in this field, especially in the context of live-cell imaging and super-resolution microscopy (SRM). SRM techniques, such as structure illuminated microscopy (SIM), stimulated emission depletion microscopy (STED), photoactivation localization microscopy (PALM), stochastic optical reconstruction microscopy (STORM) or DNA point accumulation for imaging in nanoscale topography (DNA-PAINT) have been recently developed, allowing to bypass the diffraction limit barrier of conventional optical microscopy [162,163,164,165,166,167]. Along with these developments, there is a need for improving labeling strategies, in order to better visualize molecules in their physiological state. Staining with conventional antibodies is currently the most popular modality to image antigens on fixed cellular or tissue samples, which is, however, not compatible with common live-cell imaging approaches. In addition, antibody dimension (150 kDa, 10–15 nm) can interfere reducing imaging resolution, as they displace the fluorophore from the target, introducing the so-called “linkage error” [168]. Being about 10 times smaller than conventional Abs, Nbs represent the desired tool for both live cell imaging and SRM (Figure 3). To visualize dynamic processes in live cells, proteins of interest can be fused to fluorescent proteins (FPs) or self-labeling enzymes: however, the large dimension of these protein tags (20–25 kDa) may significantly interfere with protein function and localization [169,170]. To visualize endogenous targets, intracellularly functional binding molecules (intrabodies), which are based on single-chain variable fragment (scFv) generation, have been recently developed [171,172].

Nbs are emerging as interesting tools to study intracellular pathways in live cell microscopy [173]. To visualize endogenous molecules, Nbs are genetically fused to FPs and inserted as DNA-constructs, called chromobodies, in living cells [70]. A first anti-GFP chromobody was used to identify different GFP-labeled constitutive cell proteins, including components of the nuclear lamina, chromatin and cytoskeleton [70]. Subsequently, different chromobodies have been generated and successfully used to dynamically study cytoskeleton remodeling, as well as nuclear components [173,174,175,176]. For instance, an anti-vimentin chromobody was generated to evaluate cytoskeletal modification and vimentin fiber formation in epithelial-to-mesenchymal transition, induced by TGFβ, in a lung cancer cell model [177,178]. Similarly, an anti-PARP1 chromobody was used to detect PARP1 recruitment at the site of DNA-damage, while native proliferating cell nuclear antigen (PCNA) targeting chromobody allowed the time-lapse quantitative analysis of DNA replication and S phase progression in human cells [179]. In addition, chromobodies can provide information related to functional changes within the cells, by fusing Nbs with fluorescent sensors for Ca^2+^ concentration or pH [180,181]. However, although chromobodies are considered extremely useful tools for live cell imaging, signal related to the unbound probe represents a limitation due to background fluorescence: therefore, conditionally stable chromobodies or enhancer Nbs have been subsequently generated [182,183,184]. More recently, an alternative method for nanobody delivery into living cells, based on in vitro-transcribed (IVT) mRNA, has been applied: anti-GFP Nb expressed via IVT mRNA were detected already 3h after transfection, in contrast to the 24 h required upon DNA delivery [185]. An interesting application of chromobodies has been recently developed for in vivo super-resolution microscopy: actin-chromobody fused to far-red fluorescent protein was successfully applied to visualize neuronal actin plasticity in living mouse brain, indicating the possibility to use chromobody not only for in vitro live cell imaging but also in intravital microscopy settings [186].

In SRM, the first Nb application has been performed by SIM, using an ATTO488^®^-labeled anti-GFP Nbs [187]. Subsequently, anti-GFP and anti-RFP Nbs, conjugated with different dyes, have been developed and applied in different SRM techniques, thus minimizing linkage error occurrence and combining the high photon yield of organic dyes with the molecular specificity of genetic tagging [188,189,190,191,192].

In addition to FPs, other tags can be used to generate fusion proteins and then applied as targets for Nb imaging. A short and inert BC2 peptide-tag and the corresponding bivalent Nbs (bivBC2-Nb) were used to visualize vimentin by high-quality direct-STORM imaging [193]. Similarly, a 15 amino acid peptide ALFA, in combination with a specific ALFA-targeting Nb, has been generated and used to visualize intracellular ALFA-tagged protein by STED microscopy [194]. More recently, another short peptide tag—PepTag—recognized by a specific Nb, has been applied for the direct immunofluorescence staining of tagged protein in vitro. In addition, a fluorescent Pep-chromobody was generated for live cell imaging applications [195].

Fluorescence-labeled Nbs can be also used to directly target native intracellular structure [189]. In a recent publication, a Nb-based fluorescent reporter recognizing the human α-Synuclein (hαSyn) was developed [196]. HαSyn aggregation and spreading are hallmarks of several neurodegenerative diseases: the Nb NbSyn87, conjugated with a fluorescent reporter, was able to specifically detect and analyze the amount of intracellular hαSyn [196]. Thanks to their smaller dimension, Nbs were able to visualize and resolve structures undetectable with conventional Abs—in a recent study, Nb targeting SNAP-25 and Syntaxin 1A, two plasma membrane proteins, involved in the release of neurotransmitters at synapses, were visualized by SRM and revealed a large expression of SNAP-25 and Syntaxin 1A outside the synapses, which were barely detectable with conventional Abs [197].

Finally, Nbs can be used as an alternative to standard secondary Abs (Figure 3). Using indirect immunostaining for antigen detection, the primary-secondary Ab complex can increase the apparent dimension of the target molecules or introduce a 10-20 nm localization bias [188,198]. The use of fluorescent-labeled Nbs as secondary antibody can reduce the distance between the antigen and the dye, thus limiting the linkage error. A toolbox of Nbs against mouse IgG subclasses and rabbit IgG has been generated and used for STORM imaging of microtubules, showing a reduced label displacement compared to conventional secondary Abs [199]. More recently, it has been demonstrated that secondary Nbs, in addition to increase spatial resolution and penetrate deeper in thick tissues, can be incubated with the correspondent primary Abs to form a complex directly used for staining, thus reducing experimental time [200].

## 7. Conclusive Remarks

Since their discovery and description in the nineties, the interest in the potential application of Nbs in preclinical and clinical studies has progressively grown. The extraordinary features of Nbs make them an extremely versatile tool for different imaging applications. Thanks to their small size and high tissue penetration, Nbs are ideally suited for non-invasive in vivo molecular imaging application. Accordingly, several Nb-based probes have been generated, targeting molecules whose role has been clearly established in several experimental models of diseases, including cancer, atherosclerosis and neuronal disorders. Preclinical studies have widely demonstrated the great potential of Nbs as molecular imaging tracers. Moreover, several clinical trials are running to evaluate their use as diagnostic agents in humans, especially in oncology. Although their rapid clearance represents an advantage for in vivo imaging, resulting in a decreased blood circulation and increased signal-to-noise ratio, this aspect could be a limitation for their use as therapeutic agents. Nevertheless, the possibility to be easy engineered allows several structural modifications, enhancing their retention and accumulation in target tissues, thus making Nb-based therapy another interesting field of research.

In addition to non-invasive imaging application, Nbs are extremely suited for microscopy techniques, especially for live cell imaging and super-resolution modalities. Although only a limited number of Nbs is available for SRM, several studies clearly underline their great contribution in improving the spatial resolution of the imaging analysis, pushing even forward the possibility to visualize the structure, localization and function of different molecules at nanoscale level.

The literature about the use of Nbs as preclinical imaging agents is vast and clearly indicates the high versatility of these molecules, whose applications range from the macroscopic to nanoscopic investigation of biological processes. Moreover, the route for the translation of some Nb-based methodologies in clinical settings has already started, providing an innovative and precise tool for the diagnosis and treatment of several human diseases.
